# The Role of Backbone Hydration of Poly(N-isopropyl acrylamide) Across the Volume Phase Transition Compared to its Monomer

**DOI:** 10.1038/s41598-017-17272-7

**Published:** 2017-12-05

**Authors:** Moritz H. Futscher, Martine Philipp, Peter Müller-Buschbaum, Alfons Schulte

**Affiliations:** 1 Technische Universität München, Physik-Department, Lehrstuhl für Funktionelle Materialien, James-Franck-Str. 1, 85748 Garching, Germany; 2 University of Central Florida, Department of Physics and College of Optics and Photonics, 4111 Libra Drive, Orlando, FL 32817–2385 United States

## Abstract

Thermo-responsive polymers undergo a reversible coil-to-globule transition in water after which the chains collapse and aggregate into bigger globules when passing to above its lower critical solution temperature (LCST). The hydrogen bonding with the amide groups in the side chains has to be contrasted with the hydration interaction of the hydrophobic main-chain hydrocarbons. In the present investigation we study molecular changes in the polymer poly(N-isopropyl acrylamide) (PNIPAM) and in its monomer N-isopropyl acrylamide (NIPAM) in solution across the LCST transition. Employing Fourier-transform infrared spectroscopy we probe changes in conformation and hydrogen bonding. We observe a nearly discontinuous shift of the peak frequencies and areas of vibrational bands across the LCST transition for PNIPAM whereas NIPAM exhibits a continuous linear change with temperature. This supports the crucial role of the polymer backbone with respect to hydration changes in the amide group in combination with cooperative interactions of bound water along the backbone chain.

## Introduction

Water-soluble polymers continue to attract increasing interest due to a wide range of applications in biotechnology and medicine such as drug delivery, wound healing, mineral processing, and bio-sensing^1–6^. An intriguing feature in stimuli-responsive polymers in solution or in gels is their ability to undergo a reversible phase transition at a critical temperature^7–14^. During this transition large amounts of water can be released or absorbed over a very narrow temperature range. Therefore, stimuli-responsive polymers in the form of hydrogel networks and as thin films can serve as actuators for thermoresponsive surfaces^15^, as thin film sensors^16^, and as nanostructured gratings for cell adhesion^17^. Poly(N-isopropyl acrylamide) (PNIPAM) is a prototype polymer which can undergo a sharp demixing transition at the lower critical solution temperature (LCST) in an aqueous environment. It exhibits environmental sensitivity to both temperature and pressure changes, which makes it an excellent candidate for fundamental studies of the hydration properties^18–23^.

In a PNIPAM aqueous solution there is polymer-bound water in addition to the bulk water acting as the solvent. Part of the bound water interacts via hydrogen bonds with some C=O or N-H groups; other interactions can take place with the hydrophobic moieties such as the methyl groups or the main-chain hydrocarbons^24–26^. PNIPAM possesses a particularly sharp demixing transition in an aqueous environment with a LCST near to 32 °C^27,28^. The high temperature sensitivity has been suggested to be caused by the cooperative dehydration of polymer molecules during heating^29^. The phase separation across the LCST transition leads to a partial dehydration of the individual PNIPAM chains while their conformation changes from coiled to globular^30,31^. During the coil-to-globule transition, intra- and interchain hydrogen bonds form between the amide groups, which are accompanied by a decrease in the amount of bound water^32–34^. The transition temperature of short PNIPAM oligomers was shown to be dependent on the molecular weight^35,36^. Molecular dynamics simulations even suggest that oligomers with a chain length smaller than 8 monomer units do not exhibit a coil-to-globule transition^37^. Furthermore, an effect on the phase behavior of PNIPAM has been observed due to different solution compositions^38^. Bischofberger *et al.* ^39^ suggest that hydrophobic hydration is strongly determined by the mean energetic state of water that is tuned by the addition of an organic solute. However, despite many studies on phase separating PNIPAM solutions the details of the dehydration process and the role of the polymer backbone across the LCST is still not well understood^40,41^. In particular, there is a lack of studies comparing hydration changes in the polymer with its monomer.

Vibrational spectroscopy provides a versatile *in-situ* approach to probe molecular interactions across the LCST transition for polymers with different chemical structures. Infrared spectroscopy long has been used to study aqueous PNIPAM solutions^42,43^. It has been employed to reveal differences in the microenvironment of the individual chemical groups across the PNIPAM phase transition. For example, Katsumoto *et al*. observed large changes in the amide bands of aqueous PNIPAM solutions with temperature-dependent infrared spectroscopy measurements, and attributed these to the formation of intermolecular hydrogen bonds between C=O and N-H of neighboring amide groups^44^. Maeda *et al*. found that the C-H stretching IR bands shift to lower wavenumbers during the heating process due to the dehydration of the isopropyl side chain and the main chain^25^. These authors also suggested that part of the amide groups of PNIPAM do not form intra- or intermolecular hydrogen bonds between C=O and N-D, but instead form hydrogen bonds with water in the demixed state of the solution. Cheng *et al*. showed that in dilute aqueous PNIPAM solutions the disruption of hydrogen bonds between C=O and N-D is reversible during a coil-to-globule-to-coil transition path^45^. They also noted that a heating-and-cooling cycle can lead to a hysteresis due to remaining hydrogen bonds during the cooling process. In another infrared spectroscopic study, Sun *et al*. propose a sequential dehydration process where the CH_3_ groups dehydrate first when the temperature rises across the transition, followed by a transition in the hydrogen bond network^26^.

In the present work we investigate the role of the polymer backbone in hydration changes that are crucial for the LCST transition. It should be noted that in contrast to peptides the amide group is located in the side chain. High resolution infrared spectra are measured, and spectral changes in the polymer are compared with those in its monomer to assess the role of cooperativity along the polymer chain. The vibrational spectra allow to distinguish hydrophobic hydration of the hydrocarbons in the side chain and the backbone from the hydrogen bonding with the amide groups in the side chains. In addition to the Amide I we probe the Amide II by using H_2_O as a solvent. Upon deuteration the intensity of the Amide II band at near 1560 cm^−1^ disappears. Infrared spectra of both PNIPAM polymer and NIPAM monomer are first presented over the complete frequency range from 1000 to 4000 cm^−1^. The spectra are deconvoluted in the regions of the main spectral markers. We demonstrate significant differences in the shift of peak frequencies between the polymer and its monomer. In particular, step-like changes in the temperature dependence of vibrational frequencies are absent in the NIPAM monomer solution indicating the absence of a sharp transition. The weak observed temperature dependence of the vibrational frequencies may be attributed to thermal expansion. In contrast, we observe nearly discontinuous changes in the band areas and frequencies in the PNIPAM polymer spectra at the phase transition, with very small hysteresis between heating and cooling. Finally, we discuss detailed changes in the side chains (Amide I and Amide II bonds) versus the backbone (C-H groups) and their role in the transition.

## Results and Discussion

**Figure 1:**
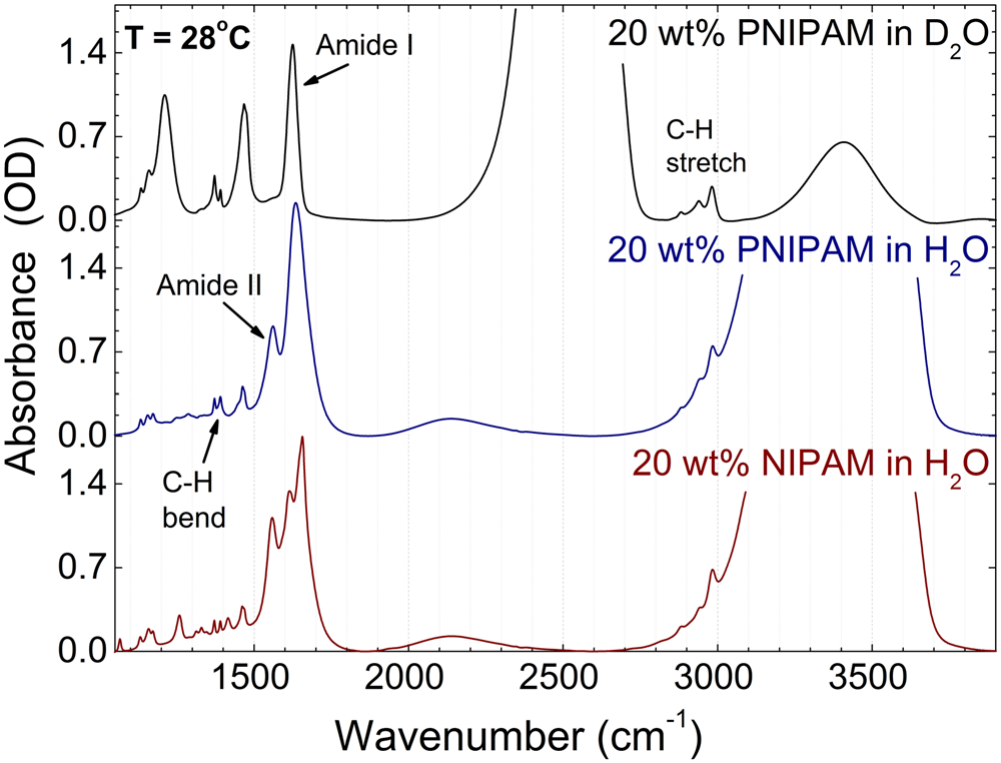
FTIR spectra of PNIPAM in 20 wt% D_2_O solution, PNIPAM in 20 wt% H_2_O solution and NIPAM in 20 wt% H_2_O solution measured at 28 °C.

In order to study the difference between PNIPAM and its monomer NIPAM, the temperature dependence of their characteristic spectral bands is investigated with Fourier-transform infrared (FTIR) spectroscopy. The infrared spectra of PNIPAM in both D_2_O and H_2_O solutions and of NIPAM in H_2_O solution at the temperature of 28 °C are shown in Fig. 1 over the frequency range from 1000 to 4000 cm^−1^. We can distinguish the following main spectral bands: The broad absorptions around 3400 and 2500 cm^−1^ correspond to the O-H and O-D stretch vibrations, respectively^46^. The bands assigned to C-H deformation modes are observable between 1350 and 1450 cm^−1^. The vibrational bands due to C-H groups are discernible in the range 2850 and 3000 cm^−1^ while the bands between 1500 and 1750 cm^−1^ are assigned the Amide I and Amide II vibrations^25,26,45^. The Amide I band arises from the C=O stretching modes that are localized on the carbonyl (~80% potential energy distribution) with some contribution from C-N stretching (~20%) vibrations^47–49^. The Amide II band has been assigned to a combination of N-H in-plane bending (~60%) and C-H stretching (~40%) vibrations. The Amide I band is most sensitive to secondary structure changes whereas the Amide II band is most sensitive to H or D exchange. The Amide I band at 1546 cm^−1^ disappears upon deuteration while the deuterated Amide II’ band overlaps with the CH_3_ bending vibration. To investigate changes in the Amide II spectral bands, PNIPAM and NIPAM were dissolved in H_2_O.

**Figure 2:**
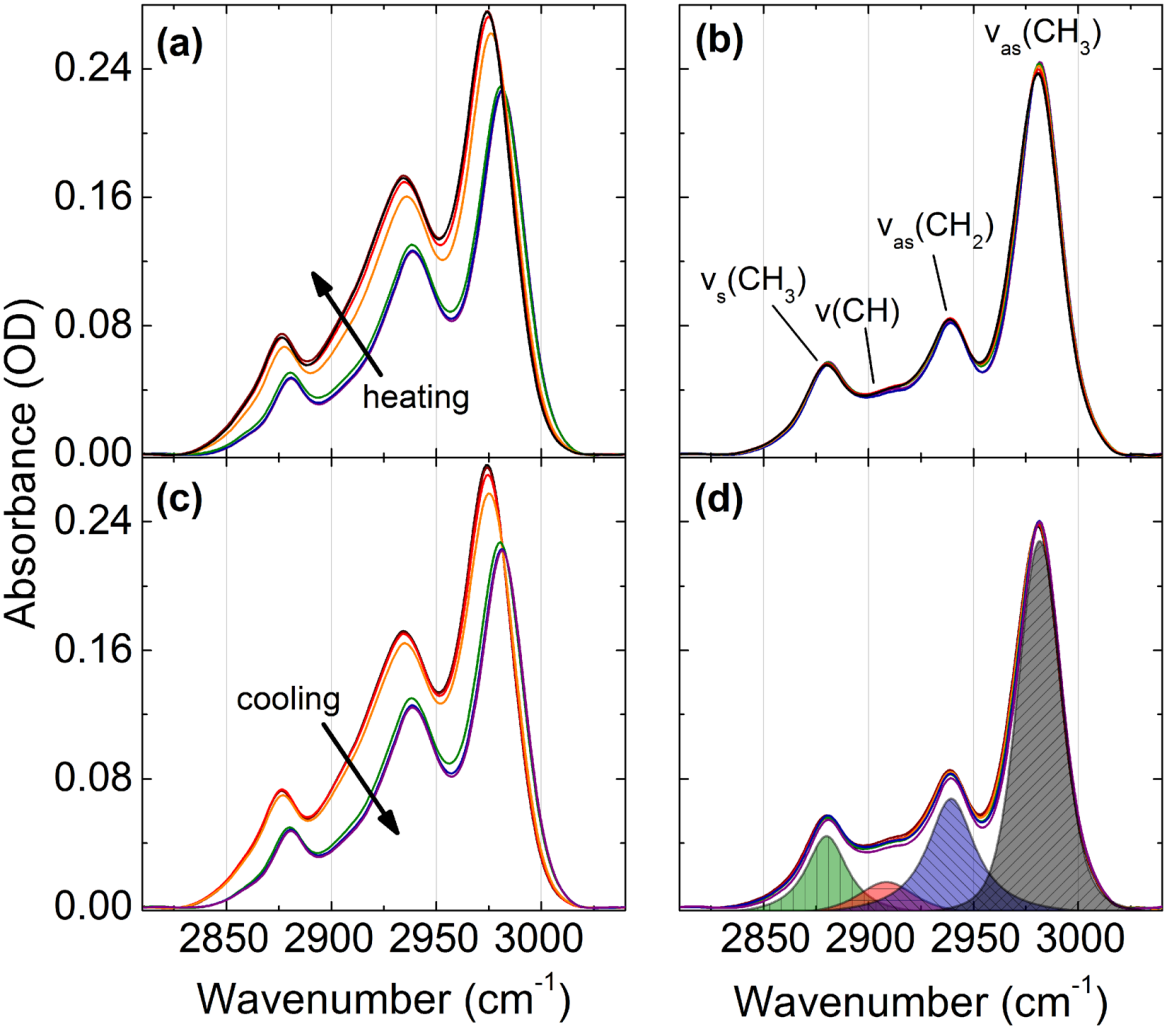
Absorption bands of the vibrational C-H groups of PNIPAM (**a**,**c**) and NIPAM (**b**,**d**) in 20 wt% H_2_O solution while heating (**a**,**b**) and cooling (**c**,**d**).

Infrared absorption spectra of PNIPAM and NIPAM in a 20 wt% H_2_O solution were acquired during heating and cooling cycles. The background corrected FTIR spectra are shown in Fig. 2 after subtracting the broad O-H stretching band (see Supplementary Figure S1).

The four sub bands of the C-H absorption are centered at about 2981, 2938, 2911, and 2877 cm^−1^ at room temperature. These are attributed to asymmetric and symmetric vibrations of the methyl groups and labeled as v_as_(CH_3_), v_as_(CH_2_), v(CH), and v_s_(CH_3_), respectively^50^. At the LCST transition we observe an almost step-like frequency shift and a change in absorbance of PNIPAM that is absent with NIPAM.

**Figure 3:**
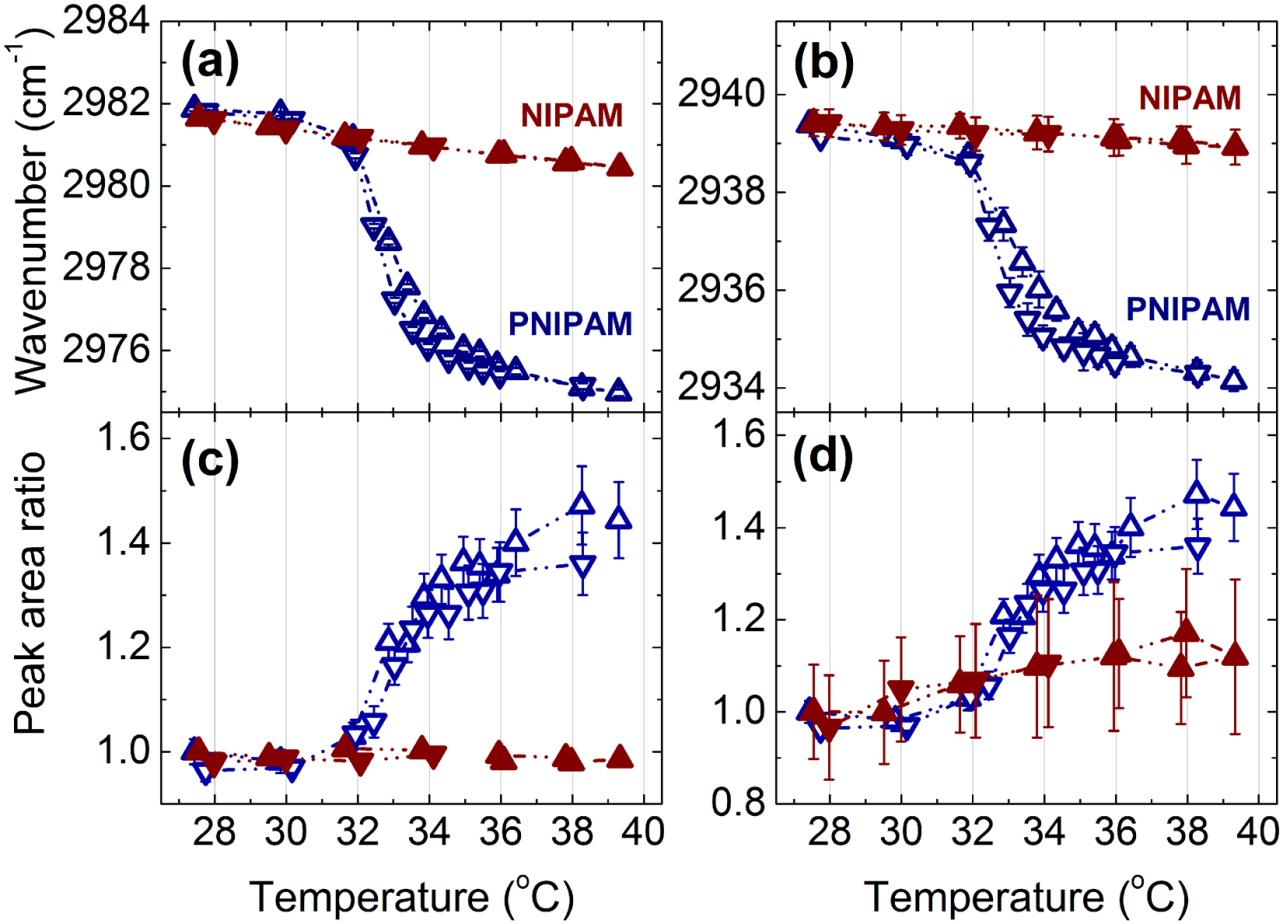
Peak position (**a**,**b**) and peak areas (**c**,**d**) of v_as_(CH_3_) (**a**,**c**) and v_as_(CH_2_) (**b**,**d**) of PNIPAM and NIPAM in 20 wt% H_2_O solution as a function of temperature measured with FTIR spectroscopy. The upturned triangles indicate the heating process and the downward triangles show the cooling process.

In order to determine the peak positions and areas four Voigt line-shapes are fitted to the vibrational bands assigned to the C-H groups. Voigtian line shapes consist of a Gaussian distribution of Lorentzian lines and are well suited to describe inhomogeneous broadened lines in soft matter^51^. The temperature dependence of the peak position and of the peak areas of v_as_(CH_3_) and v_as_(CH_2_) during a heating-cooling cycle are depicted in Fig. 3. The v_as_(CH_3_) vibration is assigned to symmetric methyl bending vibrations of the side chain of PNIPAM, and the v_as_(CH_2_) vibration is assigned to the main chain of PNIPAM^26^. In the 20 wt% PNIPAM solution in H_2_O, the peak positions of the vibrational C-H bands shift to lower wavenumbers across the LCST transition upon heating. Simultaneously, increases in the peak areas of the C-H vibrational bands are observed across the LCST transition upon heating. The change in peak position and area of the vibrational C-H groups can be attributed to the cooperative hydration effect in an aqueous environment while changes in intensity reflect changes in molar absorption^52–55^. The step-like behavior of both peak position and area of the C-H groups of PNIPAM therefore indicates a conformational change from coil below to globule accompanied by a dehydration of the C-H groups above the LCST transition. It is accompanied by the formation of a cage-like structure of water molecules surrounding the polymer^56^. Furthermore, strong increases in the full width at half maxima of the Voigtian line profiles^57^ are observed across the LCST transition while heating (see Supplementary Information, Figures S2–S3). This indicates a more heterogeneous polymer ensemble in the demixed phase. The changes in both wavenumber and peak area are reversible with a minor hysteresis in temperature of about 1 °C. The hysteresis may be due to the formation of some additional intra- and interchain hydrogen bonds in the collapsed state which cannot be easily removed in the cooling process. The reversible changes in peak areas for both PNIPAM and NIPAM with temperature are completely reproducible. In terms of the measurement, this indicates by the high thermal homogeneity and stability of the sample environment. Discontinuous changes in frequency and area of the C-H bands with temperature are absent in the 20 wt% NIPAM. The approximate linear changes in peak position of the C-H groups of NIPAM can be attributed to thermal expansion^54^. Note that measurements of CH_2_ groups will probe the backbone methylene dynamics, which is absent in NIPAM.

**Figure 4:**
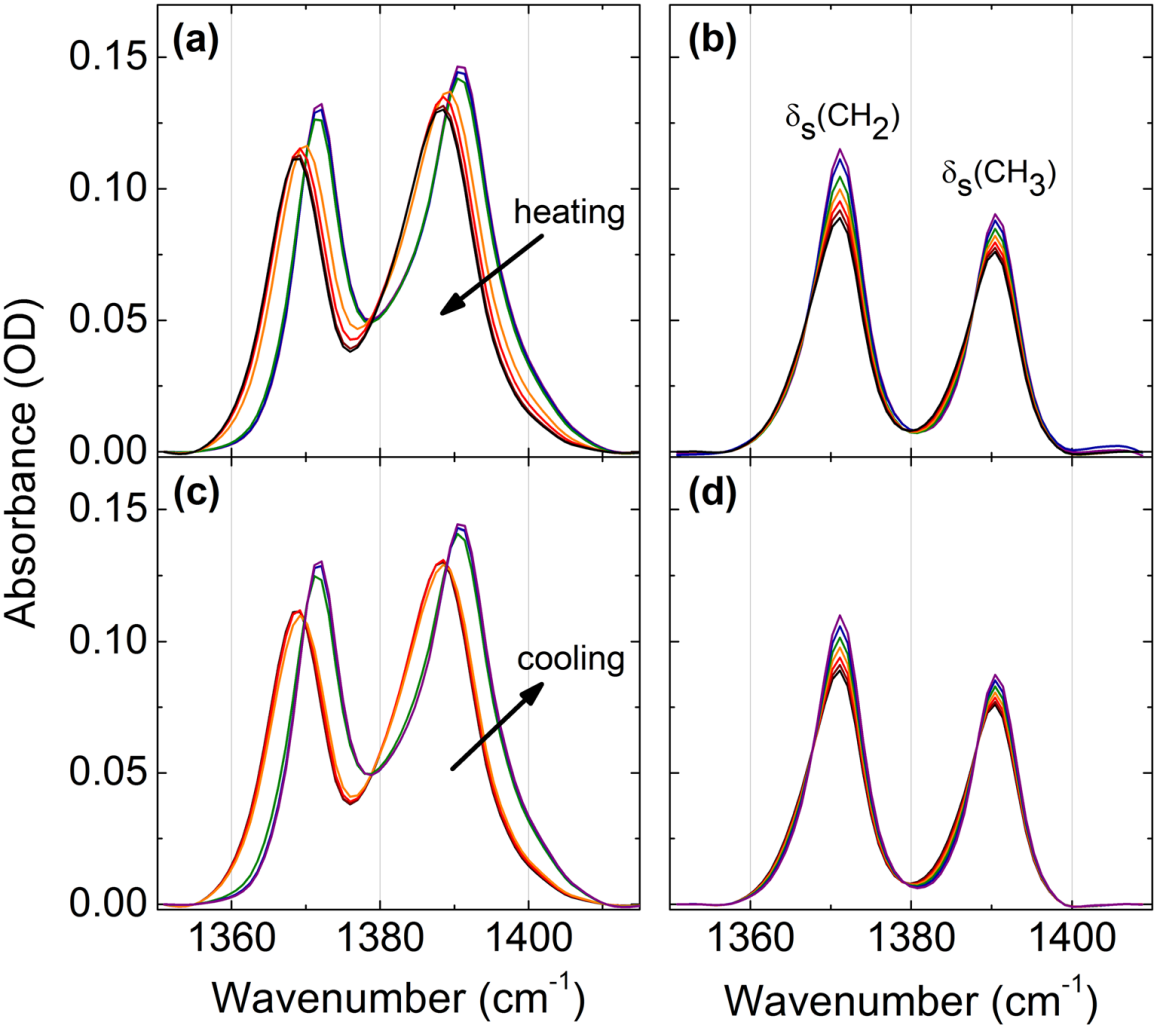
Absorption bands of the C-H deformation modes of PNIPAM (**a**,**c**) and NIPAM (**b**,**d**) in 20 wt% H_2_O solution while heating (**a**,**b**) and cooling (**c**,**d**).

**Figure 5:**
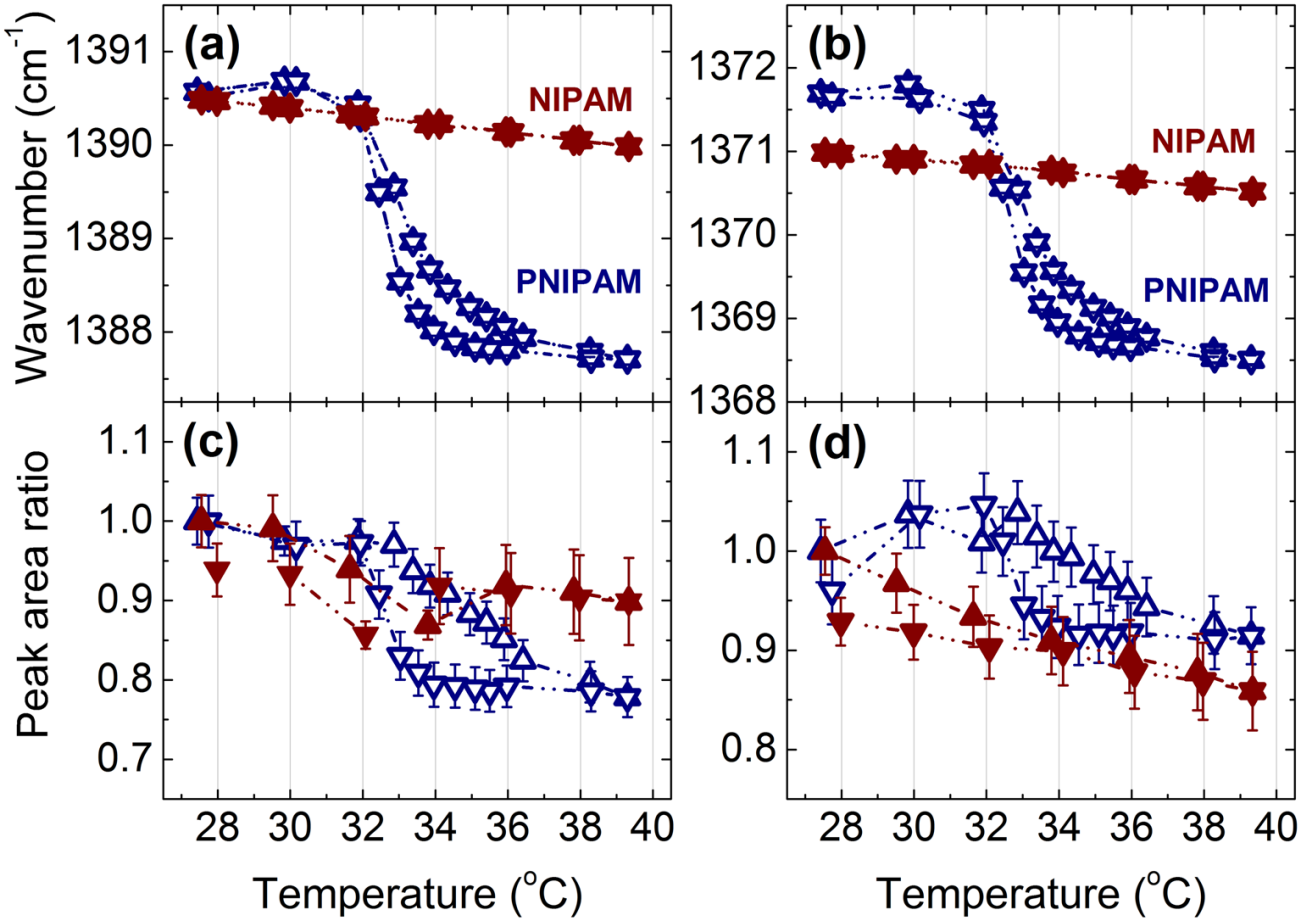
Peak position (**a**,**b**) and peak areas (**c**,**d**) of δ_s_(CH_3_) (**a**,**c**) and δ_s_(CH_2_) (**b**,**d**) of PNIPAM and NIPAM (20 wt%) in H_2_O solution as a function of temperature. The upturned triangles indicate the heating process and the downward triangles show the cooling process.

The spectral bands assignable to the CH_2_ and CH_3_ deformation modes of PNIPAM and NIPAM in a 20 wt% solution in H_2_O for both increasing and decreasing temperature runs are shown in Fig. 4. The two components centered near 1390 and 1371 cm^−1^ at room temperature are assigned to the symmetric methylene deformation modes^50^. These are respectively labeled as δ_s_(CH_3_) and δ_s_(CH_2_) and correspond to vibrations in the sidechain and the backbone. At the LCST transition we observed a distinct change in wavenumber and peak area of the deformation modes of PNIPAM, but not for NIPAM. The spectral bands assigned to the C-H deformation modes were deconvoluted with two Voigtian line profiles. The resulting peak positions and areas of δ_s_(CH_3_) and δ_s_(CH_**2**_) during a heating-cooling cycle are shown in Fig. 5. The full width at half maxima of the Voigtian line profiles as a function of temperature are shown in Supplementary Figures S4–S5. Similar to the observed changes of the C-H groups spectra, the deformation modes in PNIPAM exhibit an almost step-like behavior in wavenumber, peak area, and full-width at half maximum across the LCST transition. On the other hand, in NIPAM we observe almost continuous and approximately linear changes with temperature.

**Figure 6:**
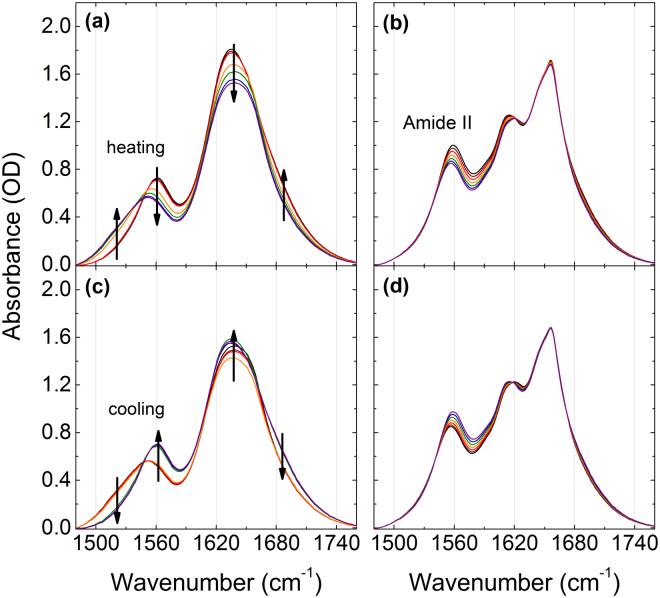
Absorption bands of the Amide I and the Amide II group of PNIPAM (**a**,**c**) and NIPAM (**b**,**d**) in 20 wt% H_2_O solution while heating (**a**,**b**) and cooling (**c**,**d**).

Figure 6 displays the Amide I and II bands measured during runs with increasing and decreasing temperature showing different behavior in PNIPAM and NIPAM. The absorption bands near 1649 and 1624 cm^−1^ are attributed to C=O hydrogen bonded with N-D and C=O hydrogen bonded with H-O-H, respectively^58^.

The ‘peptide group’ vibrations have been intensively studied in polypeptides and proteins^47^. In the simplest molecular model for the peptide linkage, N-Methylacetamide, it was found that it is vibrationally coupled with its hydrogen-bonded water molecules^59–61^. In polyalanine peptides with α-helical and polyproline II conformations negligible coupling occurs for the Amide II vibrations between adjacent peptide bonds^59–61^. Here we treat the amide and CH_3_ vibrations as independent. The change in peak intensity is thus due to the coil-to-globule transition where the C=O groups dehydrate and new hydrogen bonds between C=O and N-H of neighboring amide groups are formed. Quantum chemical calculations have been carried out by Koyama *et al*. for NIPAM n-mers with an isotactic stereo sequence^62^. These authors suggest that the Amide I band near 1625 cm^−1^ arises from a helical structure formed by the isotactic stereo-sequences in the PNIPAM main chain with the aid of intramolecular C=O•••N-H hydrogen bonding. Furthermore, a decrease in intensity near 1560 cm^−1^ and an increase in intensity near 1530 cm^−1^ can be seen across the LCST. The bands near 1560 and 1530 cm^−1^ are attributed to N-H hydrogen bonded with O-H_2_ and N-H hydrogen bonded with O = C, respectively^25^. This indicates that N-H groups dehydrate above the LCST and new hydrogen bonds are formed between N-H and O = C of neighboring amide groups. On the other hand, corresponding bands for NIPAM exhibit approximately linear changes in peak position and area with temperature for both the Amide I and Amide II bands, which is attributed to thermal expansion and to the change in molar absorption, respectively^52^.

While intensity changes in the Amide I’ band marking the LCST transition have been analyzed previously in D_2_O solution^21,26^, the transition can also be discerned from frequency shifts in both the Amide I and the Amide II bands in H_2_O solution. However, a detailed deconvolution of the amide bands is challenging due to the underlying bending vibration of the H_2_O solvent at 1643 cm^−1^ ^46^. Ahmed *et al*. have carried out resonance Raman experiments with deep UV excitation, thereby enhancing the amide bands over the solvent^49^. They observed frequency shifts of 11 cm^−1^ and 7 cm^−1^ between 30 and 45 °C for Amide I and Amide II, respectively.

**Figure 7:**
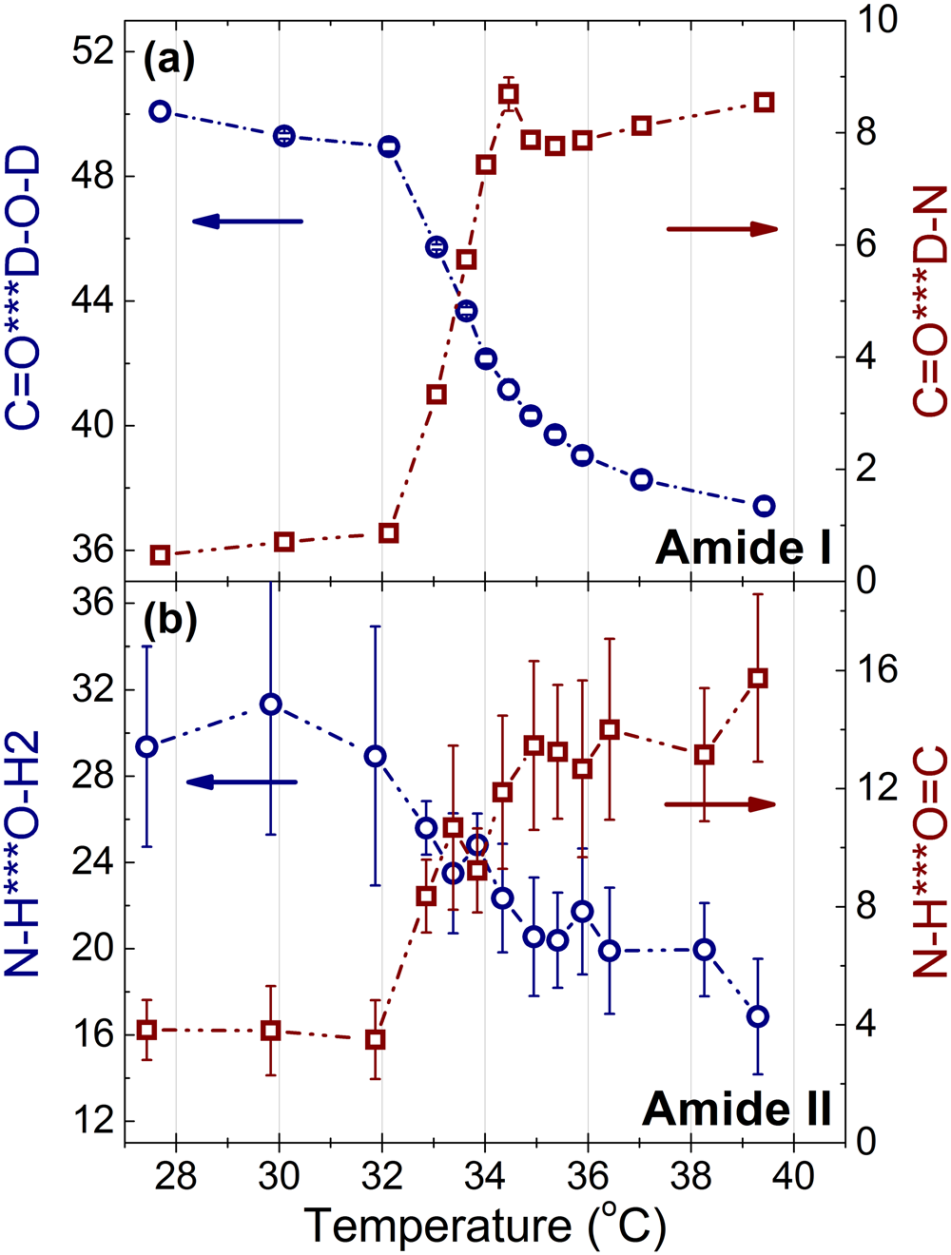
Temperature evolution of the PNIPAM Amide I band in a 20 wt% D_2_O solution (**a**) and the PNIPAM Amide II band in a 20 wt% H_2_O solution (**b**). The y-axis shows the peak areas of the Amide I (C=O hydrogen bonded with D-O-D centered near 1624 cm^−1^ and C=O hydrogen bounded with D-N centered near 1649 cm^−1^) and the Amide II (N-H hydrogen bonded with O-H_2_ centered at about 1560 cm^−1^ and N-H hydrogen bonded with O = C centered at about 1530 cm^−1^).

In order to analyze the dehydration of the C=O groups and the N-H groups, Voigt profiles were fitted to the spectral bands assigned to the Amide I and Amide II bands, respectively (see Supplementary Figures S6–S7 for details). Figure 7 displays the temperature evolution of the Amide I’ and Amide II bands of PNIPAM in 20 wt% D_2_O and 20 wt% H_2_O solution, respectively. Above the demixing transition of PNIPAM, the N-H and the C=O groups start to dehydrate, and intra- and interchain hydrogen bonds between C=O•••H-N and C=O•••D-N form. Assuming a 1:1 conversion of the C=O and the N-H groups^26^, the ratio between the peak areas of 1624 to 1649 cm^−1^, 0.67, and 1560 to 1535 cm^−1^, 0.94, respectively, yields the ratios of the molar absorptivity’s of C=O•••D-O-D and C=O•••D-N and of N-H•••O-H2 and N-H•••O = C. This suggests that only 26% of the C=O and 50% of the N-H groups dehydrate during the coil-to-globule transition and form new hydrogen bonds with D-N and O = C, respectively.

The C-H stretch and deformation modes as well as the amide bands in PNIPAM exhibit an almost step-like behavior in vibrational frequency and peak area. Changes of the spectral band parameters are discontinuous in PNIPAM, in strong contrast with NIPAM, where the band parameters change continuously with temperature. These findings indicate a crucial role of the polymer backbone with respect to hydration. Above the LCST transition the chain collapses due to cooperative hydration interactions between the water molecules hydrogen-bonded to the polymer backbone and the hydrophobic hydrocarbons. The bending of the polymer backbone then leads to the formation of intra-molecular hydrogen bonds between neighboring amide groups. The absence of these effects in NIPAM suggests that cooperative hydration in the polymer backbone is the major driving force for the phase transition in PNIPAM. This finding is supported by high-frequency dielectric relaxation data, which indicate that the number of hydrated water molecules per NIPAM molecule in aqueous solution remains constant above and below the LCST^40^. Furthermore, using ^13^C nuclear magnetic resonance (NMR) experiments, Ohta *et al*. found that chemical shifts of the amide C=O of PNIPAM in aqueous solution were decreased by ~1.5 ppm across the phase transition^63^. In accordance with our findings, they attributed this chemical shift to a reduction in the number of hydrogen bonds between the carbonyl group and water above the LCST transition. Molecular dynamics simulations in NIPAM oligomers below and above the LCST suggest that the collapse of the stable water structure in a 30-mer above the LCST leads to a coil-to-globule transition. However, it is absent in short oligomers like 3-, 5-, and 10-mer^56^.

In highly concentrated aqueous solutions of N-isopropyl propionamide (NIPPA), which is the repeating unit of PNIPAM, a broad phase transition was reported^41^. Using FTIR spectroscopy similar changes in the amide bands and in the vibrational C-H groups of PNIPAM and NIPPA were observed, indicating the same macroscopic origins of the transition^64,65^. Molecular dynamics simulations furthermore indicated that the NIPAM-solvent interaction weakens with increasing methanol concentration in a water/methanol mixture, suggesting that hydrogen bonding between the monomer units play a role in the coil-to-globule transition^66^. However, Tanaka *et al*. found that the observed reentrant coil-to-globule transition in water/alcohol mixtures arises from the competitive hydrogen bonding of the two solvents onto the polymer chain^67,68^. These results reinforce the notion that the polymer backbone is the driving force responsible for the sharp phase transition.

To further clarify the role of the polymer backbone on the coil-to-globule transition, FTIR measurements of both the monomer NIPAM and the repeating unit NIPPA of PNIPAM in solvents with different hydrogen-bonding behaviors and with different monomer concentrations would be a worthy topic for future investigations. NMR experiments probing chemical shifts of amide protons are indicators of the groups involvement with water and CO groups^63,69,70^. While beyond the scope of the present study, Hetero-Nuclear-Correlation-Spectroscopy (HSQC) experiments^71^ probing the temperature dependence of J coupling on isotopically labeled polymers could provide additional information on the involvement of amide protons^72,73^ in hydrogen bonding.

## Conclusions

In conclusion, we investigated the role of the polymer backbone in the PNIPAM phase transition through vibrational spectroscopic measurements of the polymer in comparison to its monomer. We observe significant differences in the spectral frequency shifts as a function of temperature between the polymer PNIPAM and its monomer NIPAM. Across the LCST transition peak areas and vibrational frequencies of the C-H stretch and deformation modes as well as the amide bands in PNIPAM exhibit an almost step-like behavior. On the other hand, peak areas and frequencies of the corresponding vibrational bands in NIPAM vary approximately linearly with temperature. Our findings suggest that hydration and dehydration play a crucial role for polymer backbone cooperativity and are a major driving force responsible for the sharp demixing transition.

## Methods

Poly(N-isopropyl acrylamide) (PNIPAM) and N-isopropyl acrylamide (NIPAM) were purchased from Sigma Aldrich with a purity of 97%. The molar mass of the polymer was 20–25 kg/mol corresponding to a degree of polymerization of about 200. Deuterium oxide (D_2_O, D ≥ 99.8%) was obtained from Carl Roth. PNIPAM was dissolved in Millipore water and in heavy water at a concentration of 20 wt%. NIPAM was dissolved at a concentration of 20 wt% in Millipore water. The freshly prepared solutions were shaken for at least 24 h in order to fully dissolve PNIPAM and NIPAM.

Infrared spectra were recorded with a Bruker Equinox FTIR spectrometer equipped with a DTGS detector. Data were collected at the spectral resolution of 2 cm^−1^ and signal averaging over 128 scans. The liquid sample is contained between two CaF_2_ windows (1 mm thick) within a thin doughnut-shaped Mylar spacer (Polyester, 8 μm thick). The sample, the CaF_2_ windows, and the Mylar spacer are held together by a copper holder that ensures temperature homogeneity. The sampler holder was mounted on a Cu block and connected to a circulating bath thermostat (Julabo F12) for temperature control. The temperature was measured on the sample holder with a calibrated Pt 100 thermometer at an accuracy of 0.2 °C. Variable temperature spectra were collected between 26 and 40 °C. After reaching the target temperature the sample was maintained at this temperature within 0.5 °C for 10 minutes for full equilibration. The infrared spectrum was acquired within 10 minutes. The temperature was then increased or decreased manually. Processing of the spectra, baseline correction, deconvolution and determination of wavenumbers and peak areas of the main absorption bands were done with Origin software. Curve fitting was performed using the Levenberg-Marquardt algorithm. Error bars indicate the standard error of the least-square method.

## Electronic supplementary material

Supplementary Information

## References

[1] Bawa P, Pillay V, Choonara YE, du Toit LC (2009). Stimuli-responsive polymers and their applications in drug delivery. Biomed. Mater..

[2] Pelton R (2000). Temperature-sensitive aqueous microgels. Adv. Colloid Interface Sci..

[3] Beattie DA, Addai-Mensah J, Beaussart A, Franks GV, Yeap K-Y (2014). *In situ* particle film ATR FTIR spectroscopy of poly (N-isopropyl acrylamide) (PNIPAM) adsorption onto talc. Phys. Chem. Chem. Phys..

[4] Chaterji S, Kwon IK, Park K (2007). Smart polymeric gels: Redefining the limits of biomedical devices. Progress in Polymer Science.

[5] Gil ES, Hudson SM (2004). Stimuli-reponsive polymers and their bioconjugates. Progress in Polymer Science.

[6] Qin S, Geng Y, Discher DE, Yang S (2006). Temperature-controlled assembly and release from polymer vesicles of poly(ethylene oxide)-block-poly(N-isopropylacrylamide). Adv. Mater..

[7] Heskins M, Guillet JE (1968). Solution Properties of Poly(N-isopropylacrylamide). J. Macromol. Sci. Part A - Chem..

[8] Tanaka T (1987). Mechanical instability of gels at the phase transition. Nature.

[9] Li Y, Tanaka T (1990). Kinetics of swelling and shrinking of gels. J. Chem. Phys..

[10] Tanaka F, Koga T, Kojima H, Winnik FM (2009). Temperature- and tension-induced coil globule transition of poly(N-isopropylacrylamide) chains in water and mixed solvent of water/methanol. Macromolecules.

[11] Shibayama M, Morimoto M, Nomura S (1994). Phase Separation Induced Mechanical Transition of Poly(N-isopropylacrylamide)/Water Isochore Gels. Macromolecules.

[12] Romeo G, Fernandez-Nieves A, Wyss HM, Aciernoand D, Weitz DA (2010). Temperature-controlled transitions between glass. liquid, and gel states in dense p-NIPA suspensions. Adv. Mater..

[13] Aseyev V, Tenhu H, Winnik FM (2011). Non-ionic thermoresponsive polymers in water. Advances in Polymer Science.

[14] Tanaka F, Koga T, Winnik FM (2009). Competitive Hydrogen Bonds and Cononsolvency of Poly (N-isopropylacrylamide) s in Mixed Solvents of Water/Methanol Temperature-Induced Coil-Globule. Progr Colloid Polym Sci.

[15] Cole MA, Voelcker NH, Thissen H, Griesser HJ (2009). Stimuli-responsive interfaces and systems for the control of protein-surface and cell-surface interactions. Biomaterials.

[16] Wang W (2010). Swelling and switching kinetics of gold coated end-capped poly(N-isopropylacrylamide) thin films. Macromolecules.

[17] Zhernenkov M (2015). Thermoresponsive PNIPAM Coatings on Nanostructured Gratings for Cell Alignment and Release. ACS Appl. Mater. Interfaces.

[18] Kujawa P, Winnik FM (2001). Volumetric studies of aqueous polymer solutions using pressure perturbation calorimetry: A new look at the temperature-induced phase transition of poly(N-isopropylacrylamide) in water and D2O. Macromolecules.

[19] Qiu XP, Tanaka F, Winnik FM (2007). Temperature-induced phase transition of well-defined cyclic poly(N-isopropylacrylamide)s in aqueous solution. Macromolecules.

[20] Satokawa Y, Shikata T, Tanaka F, Qiu XP, Winnik FM (2009). Hydration and dynamic behavior of a cyclic poly(N-isopropylacrylamide) in aqueous solution: Effects of the polymer chain topology. Macromolecules.

[21] Meersman F, Wang J, Wu Y, Heremans K (2005). Pressure effect on the hydration properties of poly(N-isopropylacrylamide) in aqueous solution studied by FTIR spectroscopy. Macromolecules.

[22] Meier-Koll A, Pipich V, Busch P, Papadakis CM, Müller-Buschbaum P (2012). Phase separation in semidilute aqueous poly(N-isopropylacrylamide) solutions. Langmuir.

[23] Philipp M (2014). Molecular versus macroscopic perspective on the demixing transition of aqueous PNIPAM solutions by studying the dual character of the refractive index. Soft Matter.

[24] Otake K, Inomata H, Konno M, Saito S (1990). Thermal analysis of the volume phase transition with N-isopropylacrylamide gels. Macromolecules.

[25] Maeda Y, Higuchi T, Ikeda I (2000). Change in hydration state during the coil−globule transition of aqueous solutions of poly(N-isopropylacrylamide) as evidenced by FTIR spectroscopy. Langmuir.

[26] Sun B, Lin Y, Wu P, Siesler HW (2008). A FTIR and 2D-IR spectroscopic study on the microdynamics phase separation mechanism of the poly(N-isopropylacrylamide) aqueous solution. Macromolecules.

[27] Wu C, Zhou S (1995). Thermodynamically Stable Globule State of a Single Poly(N-isopropylacrylamide) Chain in Water. Macromolecules.

[28] Wu C (1996). First observation of the molten globule state of a single homopolymer chain. Phys. Rev. Lett..

[29] Okada Y, Tanaka F (2005). Cooperative hydration, chain collapse, and flat LCST behavior in aqueous poly(N-isopropylacrylamide) solutions. Macromolecules.

[30] Kogure H, Nanami S, Masuda Y, Toyama Y, Kubota K (2005). Hydration and dehydration behavior of N-isopropylacrylamide gel particles. Colloid Polym. Sci..

[31] Ono Y, Shikata T (2006). Hydration and dynamic behavior of poly(N-isopropylacrylamide)s in aqueous solution: A sharp phase transition at the lower critical solution temperature. J. Am. Chem. Soc..

[32] Schild HG (1992). Poly(N-isopropylacrylamide): experiment, theory and application. Progress in Polymer Science.

[33] Corkhill PH, Jolly AM, Ng CO, Tighe BJ (1987). Synthetic hydrogels: 1. Hydroxyalkyl acrylate and methacrylate copolymers - water binding studies. Polymer (Guildf)..

[34] Barnes A, Corkhill PH, Tighe BJ (1988). Synthetic hydrogels: 3. Hydroxyalkyl acrylate and methacrylate copolymers: surface and mechanical properties. Polymer (Guildf)..

[35] Pamies R, Zhu K, Kjøniksen A-L, Nyström B (2009). Thermal response of low molecular weight poly-(N-isopropylacrylamide) polymers in aqueous solution. Polym. Bull..

[36] Shan J, Zhao Y, Granqvist N, Tenhu H (2009). Thermoresponsive properties of N-isopropylacrylamide oligomer brushes grafted to gold nanoparticles: Effects of molar mass and gold core size. Macromolecules.

[37] Tucker AK, Stevens MJ (2012). Study of the polymer length dependence of the single chain transition temperature in syndiotactic poly(n -isopropylacrylamide) oligomers in water. Macromolecules.

[38] Zhang G, Wu C (2001). Reentrant coil-to-globule-to-coil transition of a single linear homopolymer chain in a water/methanol mixture. Phys. Rev. Lett..

[39] Bischofberger I, Calzolari DCE (2014). De Los Rios, P., Jelezarov, I. & Trappe, V. Hydrophobic hydration of poly-N-isopropyl acrylamide: a matter of the mean energetic state of water. Sci. Rep..

[40] Ono Y, Shikata T (2007). Contrary hydration behavior of N-isopropylacrylamide to its polymer, P(NIPAm), with a lower critical solution temperature. J. Phys. Chem. B.

[41] Geukens B, Meersman F, Nies E (2008). Phase behavior of N-(isopropyl)propionamide in aqueous solution and changes in hydration observed by FTIR spectroscopy. J. Phys. Chem. B.

[42] Scarpa JS, Mueller DD, Klotz IM (1967). Slow hydrogen-deuterium exchange in a non-.alpha.-helical polyamide. J. Am. Chem. Soc..

[43] Snyder WD, Klotz IM (1975). Effect of molecular weight on hydrogen-deuterium exchange in a nonhelical polyamide. J. Am. Chem. Soc..

[44] Katsumoto Y, Tanaka T, Sato H, Ozaki Y (2002). Conformational change of poly(N-isopropylacrylamide) during the coil-globule transition investigated by attenuated total reflection/infrared spectroscopy and density functional theory calculation. J. Phys. Chem. A.

[45] Cheng H, Shen L, Wu C (2006). LLS and FTIR studies on the hysteresis in association and dissociation of poly(N-isopropylacrylamide) chains in water. Macromolecules.

[46] Venyaminov SY, Prendergast FG (1997). Water (H_2_O and D_2_O) molar absorptivity in the 1000–4000 cm^−1^ range and quantitative infrared spectroscopy of aqueous solutions. Anal. Biochem..

[47] Krimm S, Bandekar J (1986). Vibrational spectroscopy and conformation of peptides, polypeptides, and proteins. Adv. Protein Chem..

[48] Besley NA, Metcalf KA (2007). Computation of the amide I band of polypeptides and proteins using a partial Hessian approach. J. Chem. Phys..

[49] Ahmed Z, Gooding EA, Pimenov KV, Wang L, Asher SA (2009). UV Resonance raman determination of molecular mechanism of poly(n-isopropylacrylamide) volume phase transition. J. Phys. Chem. B.

[50] Sun B, Lin Y, Wu P (2007). Structure analysis of poly(N-isopropylacrylamide) using near-infrared spectroscopy and generalized two-dimensional correlation infrared spectroscopy. Appl. Spectrosc..

[51] Batty CJ, Hoath SD, Roberts BL (1976). Measurement of Lorentzian linewidths: numerical evaluation of the Voigt integral. *Nucl*. *Instruments*. Methods.

[52] Hobza P, Havlas Z (2000). Blue-Shifting Hydrogen Bonds. Chem. Rev..

[53] Joseph J, Jemmis ED (2007). Red-, blue-, or no-shift in hydrogen bonds: A unified explanation. J. Am. Chem. Soc..

[54] Demmel F, Doster W, Petry W, Schulte A (1997). Vibrational frequency shifts as a probe of hydrogen bonds: Thermal expansion and glass transition of myoglobin in mixed solvents. Eur. Biophys. J..

[55] Schmidt P, Dybal J, Trchová M (2006). Investigations of the hydrophobic and hydrophilic interactions in polymer-water systems by ATR FTIR and Raman spectroscopy. Vib. Spectrosc..

[56] Deshmukh SA, Sankaranarayanan SKRS, Suthar K, Mancini DC (2012). Role of solvation dynamics and local ordering of water in inducing conformational transitions in poly(N -isopropylacrylamide) oligomers through the LCST. J. Phys. Chem. B.

[57] Olivero JJ, Longbothum RL (1977). Empirical fits to the Voigt line width: A brief review. J. Quant. Spectrosc. Radiat. Transf..

[58] Kujawa P (2006). Impact of end-group association and main-chain hydration on the thermosensitive properties of hydrophobically modified telechelic poly(N-isopropylacrylamides) in water. Macromolecules.

[59] Chen XG, Asher SA, Krimm S, Mirkin NG, Schweitzer-Stenner R (1994). N-methylacetamide and its hydrogen-bonded water molecules are vibrationally coupled. J. Am. Chem. Soc..

[60] Chen XG, Schweitzer-Stenner R, Asher SA, Mirkin NG, Krimm S (1995). Vibrational assignments of trans-N-Methylacetamide and some of its deuterated isotopomers from band decomposition of IR, visible, and resonance raman spectra. J. Phys. Chem..

[61] Han WG, Jalkanen KJ, Elstner M, Suhai S (1998). Theoretical study of aqueous N-Acetyl-L-alanine N′-Methylamide: structures and raman, VCD, and ROA spectra. J. Phys. Chem. B.

[62] Koyama M, Hirano T, Ohno K, Katsumoto Y (2008). Molecular understanding of the UCST-type phase separation behavior of a stereocontrolled poly (N-isopropylacrylamide) in bis(2-methoxyethyl) ether. J. Phys. Chem. B.

[63] Ohta H, Ando I, Fujishige S, Kubota K (1991). A 13C PST/MAS NMR study of poly (N-isopropylacrylamide) in solution and in the gel phase. J. Mol. Struct..

[64] Lai H, Wu PA (2010). infrared spectroscopic study on the mechanism of temperature-induced phase transition of concentrated aqueous solutions of poly(N-isopropylacrylamide) and N-isopropylpropionamide. Polymer (Guildf)..

[65] Afroze F, Nies E, Berghmans H (2000). Phase transitions in the system poly(N-isopropylacrylamide)/water and swelling behaviour of the corresponding networks. J. Mol. Struct..

[66] Pang J, Yang H, Ma J, Cheng R (2010). Solvation behaviors of N-isopropylacrylamide in water/methanol mixtures revealed by molecular dynamics simulations. J. Phys. Chem. B.

[67] Tanaka F, Koga T, Winnik FM (2008). Temperature-responsive polymers in mixed solvents: Competitive hydrogen bonds cause cononsolvency. Phys. Rev. Lett..

[68] Tanaka F, Koga T, Kaneda I, Winnik FM (2011). Hydration, phase separation and nonlinear rheology of temperature-sensitive water-soluble polymers. J. Phys. Condens. Matter.

[69] Spěváček J (2009). NMR investigations of phase transition in aqueous polymer solutions and gels. Curr. Opin. Colloid Interface Sci..

[70] Sieler G, Schweitzer-Stenner R (1997). The amide I mode of peptides in aqueous solution involves vibrational coupling between the peptide group and water molecules of the hydration shell. J. Am. Chem. Soc..

[71] Diguiseppi D (2017). Probing the conformation-dependent preferential binding of ethanol to cationic glycylalanylglycine in water/ethanol by vibrational and NMR spectroscopy. J. Phys. Chem. B.

[72] Lin G, Cosimbescu L, Karin NJ, Gutowska A, Tarasevich BJ (2013). Injectable and thermogelling hydrogels of PCL-g-PEG: mechanisms, rheological and enzymatic degradation properties. J. Mater. Chem. B.

[73] Schweitzer-Stenner R (2007). Conformations of alanine-based peptides in water probed by FTIR, raman, vibrational circular dichroism, electronic circular dichroism, and NMR spectroscopy. Biochemistry.

