# Supplementary material for: The Role of Backbone Hydration of Poly(N-isopropyl acrylamide) Across the Volume Phase Transition Compared to its Monomer
Source: Sci Rep. 2017 Dec 5;7:17012. doi: 10.1038/s41598-017-17272-7 (PMC5717149; doi:10.1038/s41598-017-17272-7)
Supplement: Supplementary file 1 — Supplementary Information [file 41598_2017_17272_MOESM1_ESM.pdf]

SUPPLEMENTARY INFORMATION FOR

The Role of Backbone Hydration of Poly(N-isopropyl acrylamide) Across the Volume Phase Transition Compared to its Monomer

Moritz H. Futscher[†], *Martine Philipp*[†], *Peter Müller-Buschbaum*[†], *Alfons Schulte*[‡]*

† Technische Universität München, Physik-Department, Lehrstuhl für Funktionelle Materialien, James-Franck-Str. 1, 85748 Garching, Germany

‡ University of Central Florida, Department of Physics and College of Optics and Photonics, 4111 Libra Drive, Orlando, FL 32817-2385, United States

Corresponding Author

* Alfons.Schulte@ucf.edu

Subtracting the broad O-H stretching band

The broad O-H stretching band was subtracted by using a basis spline function fitted to the spectra in the spectral ranges between 2750 – 2830, 3020 – 3040, and 3130 – 3180 cm^{-1}. For spectra measured at 28 °C this is exemplarily shown in Figure S1.

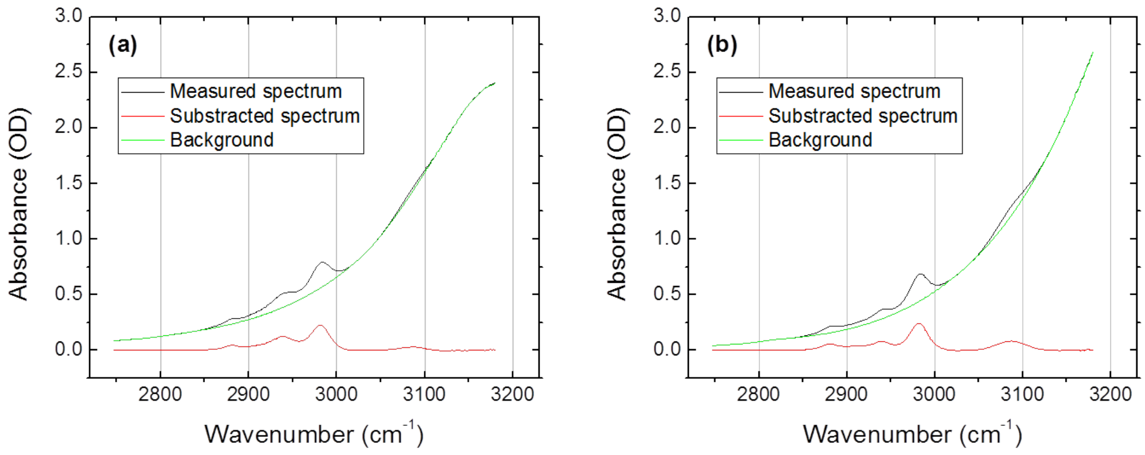

Supplementary Figure S1. Representative subtracting of the broad O-H stretching band for PNIPAM in 20 wt% H_2O solution (a) and NIPAM in 20 wt% H_2O solution (b) measured at 28 °C.

Full-width half maxima of Voigtian line profiles

The full width at half maxima (FWHM) of the fitted Voigtian line profiles as function of temperature are shown in Figure S2-S5. The FWHM are calculated using the empirical formula of Olivero and Longbothum.[1]

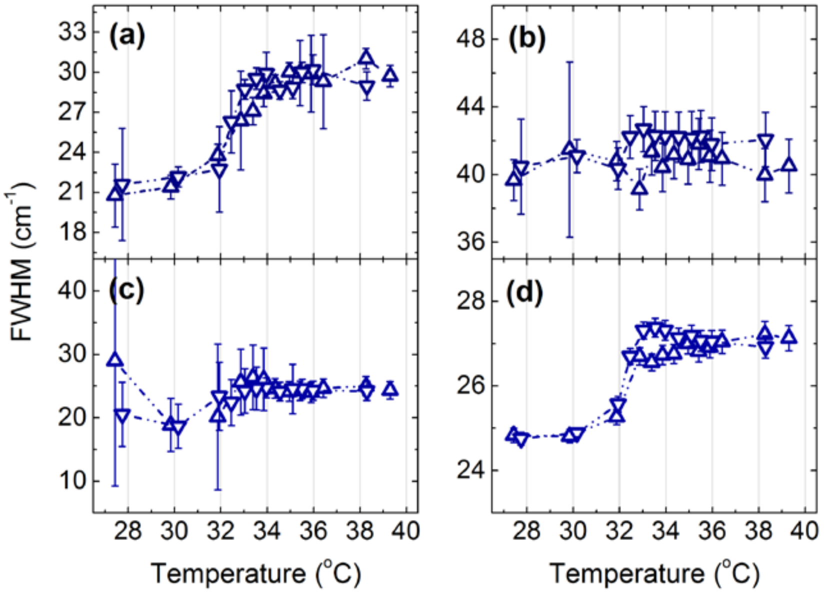

Supplementary Figure S2. FWHM of $v_s(CH_3)$ (a), $v_{as}(CH_2)$ (b), v(CH) (c), and $v_{as}(CH_3)$ (d) of PNIPAM in 20 wt% H_2O as a function of temperature measured with FTIR spectroscopy. The upturned triangles indicate the heating process and the downward triangles show the cooling process.

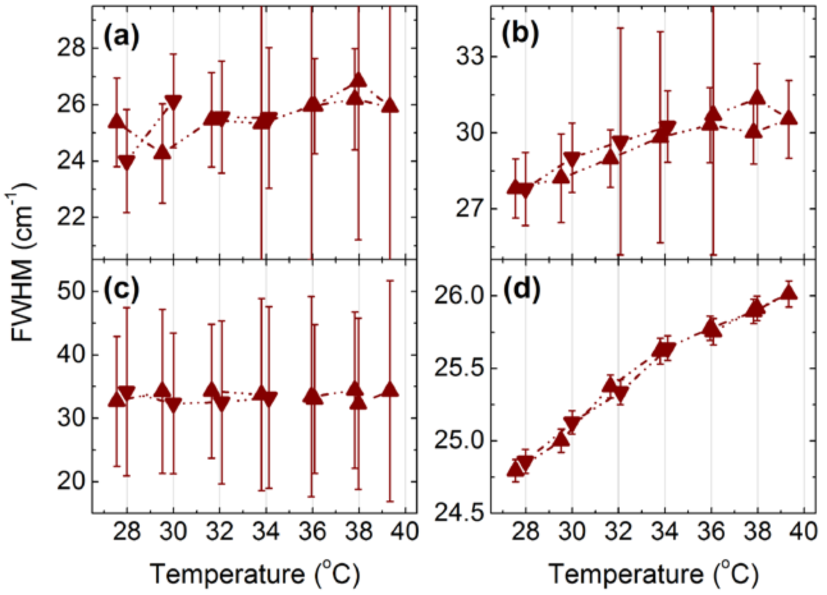

.

Supplementary Figure S3. FWHM of $v_s(CH_3)$ (a), $v_{as}(CH_2)$ (b), v(CH) (c), and $v_{as}(CH_3)$ (d) of NIPAM in 20 wt% H_2O as a function of temperature measured with FTIR spectroscopy. The upturned triangles indicate the heating process and the downward triangles show the cooling process.

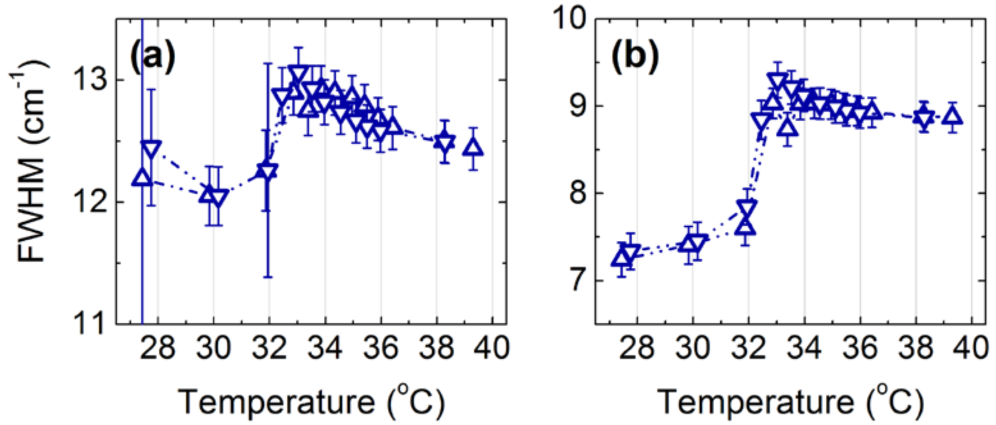

.

Supplementary Figure S4. FWHM of δ $_s$(CH_3) (a) and δ $_s$(CH_2) (b) of PNIPAM in 20 wt% H_2O as a function of temperature measured with FTIR spectroscopy. The upturned triangles indicate the heating process and the downward triangles show the cooling process.

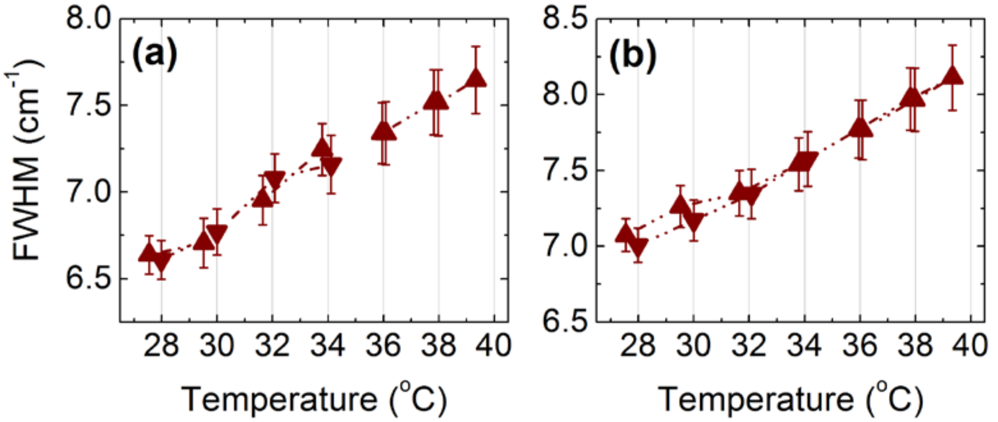

.

Supplementary Figure S5. FWHM of δ $_s$(CH_3) (a) and δ $_s$(CH_2) (b) of NIPAM in 20 wt% H_2O as a function of temperature measured with FTIR spectroscopy. The upturned triangles indicate the heating process and the downward triangles show the cooling process.

Analysis of the amide bands

In order to analyze the spectral regions assigned to the amide bands of NIPAM and PNIPAM in H_2O and D_2O solutions, background subtraction and curve fitting was performed which is exemplarily shown in Figure S6-S8.

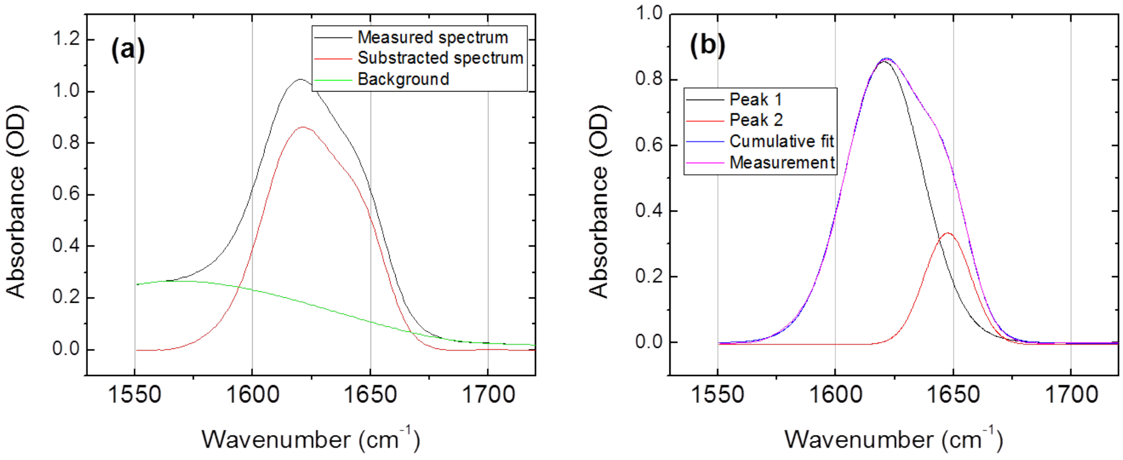

Supplementary Figure S6. Representative background subtraction (a) and deconvolution using two Voigt line profiles (b) of the spectral bands assigned to the Amide I band of PNIPAM in 20 wt% H_2O solution measured at 40 °C.

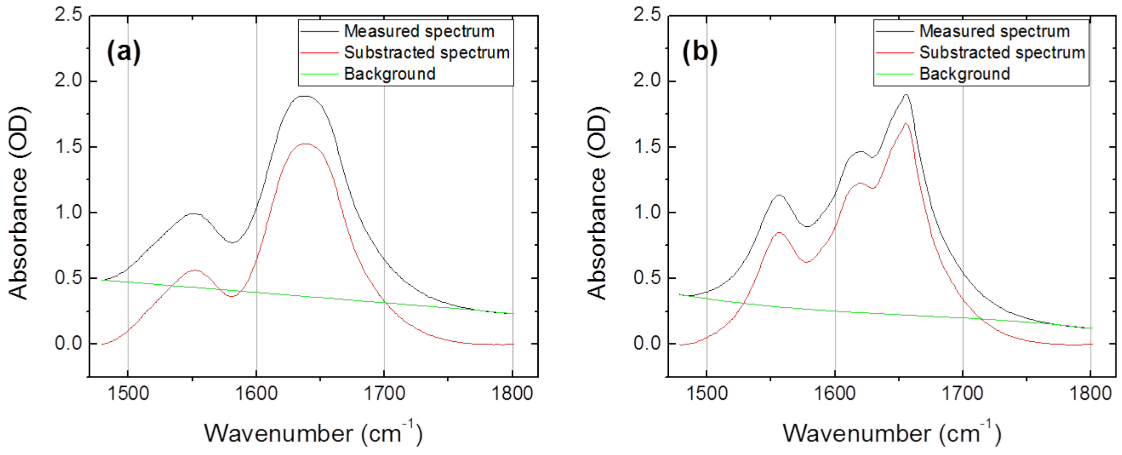

Supplementary Figure S7. Representative background subtraction of the spectral bands assigned to the Amide II bands of PNIPAM (a) and NIPAM (b) in 20 wt% D_2O solution measured at 40 °C.

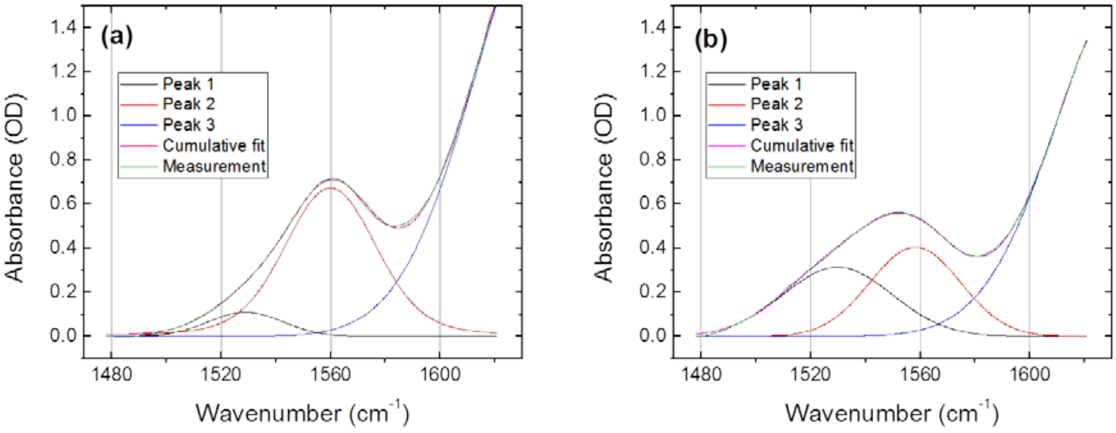

Supplementary Figure S8. Representative deconvolution using three Voigt line profiles of the spectral bands assigned to the Amide II band of PNIPAM in 20 wt% D_2O solution measured at 30 (a) and 40 °C (b).

References

1. Olivero, J. J. & Longbothum, R. L. Empirical fits to the Voigt line width: A brief review. *J. Quant. Spectrosc. Radiat. Transf.* **17,** 233–236 (1977).
